# Spatial and temporal aspects and the interplay of Grb14 and protein tyrosine phosphatase-1B on the insulin receptor phosphorylation

**DOI:** 10.1186/1478-811X-11-96

**Published:** 2013-12-18

**Authors:** Raju VS Rajala, Devaraj K Basavarajappa, Radhika Dighe, Ammaji Rajala

**Affiliations:** 1 Departments of Ophthalmology, University of Oklahoma Health Sciences Center, Oklahoma City, OK 73104, USA; 2 Departments of Physiology, University of Oklahoma Health Sciences Center, Oklahoma City, OK 73104, USA; 3 Departments of Cell Biology, University of Oklahoma Health Sciences Center, Oklahoma City, OK 73104, USA; 4 Dean A. McGee Eye Institute, 608 Stanton L. Young Boulevard, Oklahoma City, OK 73104, USA; 5 Present address: Department of Medical Biochemistry and Biophysics (MBB), Karolinska Institute (KI), C2 Scheeles Väg 2 17177, Stockholm, Sweden

**Keywords:** Grb14, PTP1B, Shp2, SRC activation, Tyrosine phosphorylation, Insulin receptor, Tyrosine kinase signaling

## Abstract

**Background:**

Growth factor receptor-bound protein 14 (Grb14) is an adapter protein implicated in receptor tyrosine kinase signaling. Grb14 knockout studies highlight both the positive and negative roles of Grb14 in receptor tyrosine kinase signaling, in a tissue specific manner. Retinal cells are post-mitotic tissue, and insulin receptor (IR) activation is essential for retinal neuron survival. Retinal cells express protein tyrosine phosphatase-1B (PTP1B), which dephosphorylates IR and Grb14, a pseudosubstrate inhibitor of IR. This project asks the following major question: in retinal neurons, how does the IR overcome inactivation by PTP1B and Grb14?

**Results:**

Our previous studies suggest that ablation of Grb14 results in decreased IR activation, due to increased PTP1B activity. Our research propounds that phosphorylation in the BPS region of Grb14 inhibits PTP1B activity, thereby promoting IR activation. We propose a model in which phosphorylation of the BPS region of Grb14 is the key element in promoting IR activation, and failure to undergo phosphorylation on Grb14 leads to both PTP1B and Grb14 exerting their negative roles in IR. Consistent with this hypothesis, we found decreased phosphorylation of Grb14 in diabetic type 1 Ins2^Akita^ mouse retinas. Decreased retinal IR activation has previously been reported in this mouse line.

**Conclusions:**

Our results suggest that phosphorylation status of the BPS region of Grb14 determines the positive or negative role it will play in IR signaling.

## Introduction

Growth factor receptor-bound protein 14 (Grb14) is an adaptor protein that is known to interact with a number of receptor tyrosine kinases and signaling molecules [1,2]. Grb14 has an inhibitory effect on receptor tyrosine kinase signaling and, in particular, on insulin receptor signaling [3]. Consistent with these findings, a genome-wide association study demonstrated that single nucleotide polymorphisms at Grb14 are strongly associated with reduced insulin sensitivity in diabetic patients [4]. While there is convincing evidence of a negative role of Grb14 in insulin signaling [5,6], experiments with *Grb14*^*-/-*^ animals have also revealed positive effects of Grb14 on receptor tyrosine kinase signaling, in a tissue specific manner [7,8].

We previously identified Grb14 in retinal tissues [9]. Interestingly, Grb14 undergoes a light-dependent intracellular translocation within rod photoreceptor neurons [8]. Light induces activation of the insulin receptor (IR) and ablation of Grb14 results in the loss of light-dependent activation of the IR [8]. In photoreceptors, Grb14 undergoes tyrosine phosphorylation by light-activated non-receptor tyrosine kinase Src, and phosphorylated Grb14 (Grb14-P) acts as a positive regulator of the IR by inhibiting PTP1B, a negative regulator of the IR [10]. Very recently, we reported that Grb14 modulates the activity of the rod cyclic nucleotide gated channel (CNG), and perhaps cGMP-phosphodiesterase in regulating rod transduction and light adaptation [11]. We also revealed that CNG channel phosphorylation is regulated by IR [12], while Grb14 regulates both IR activation and CNG channel modulation [10,11,13]. A high expression of Grb14 in myocardial tissue activates the PI3K-Akt pathway: ablation of Grb14 results in myocardial infarction and decreased PI3K/Akt activation [7]. In several models of insulin resistance, increased expression of Grb14 in adipose tissue has previously been reported [14]. Convincing evidence for a negative role of Grb14 in insulin signaling exists [15]. This evidence shows enhanced glucose tolerance and insulin sensitivity in Grb14-deficient mice [5]. Thus, our primary research question is how Grb14 achieves negative or positive roles in IR signaling. The molecular switch determining whether Grb14 will perform a particular role is unknown. Our studies suggest that the phosphorylation status of Grb14 is the key element in determining whether it will execute a negative or positive role in IR signaling.

## Results

### Effect of a phosphorylated BPS region of Grb14 on IR kinase activity

**Figure 1:**
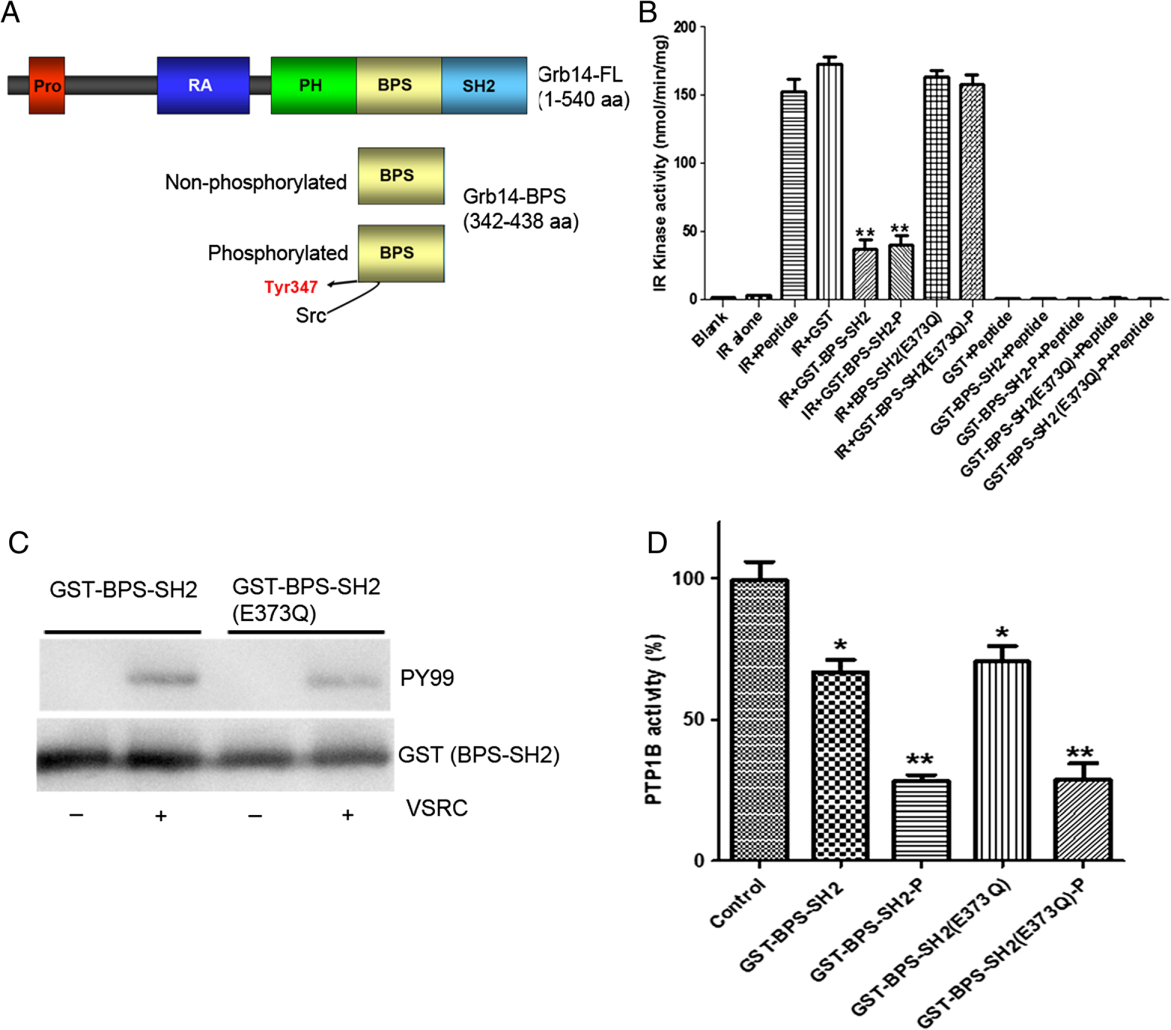
**Effect of a phosphorylated BPS region of Grb14 on IR kinase and PTP1B activity.** The domain organization of Grb14 and length of the BPS region is depicted **(A)**. IR kinase activity was measured in the presence of either GST or GST-BPS-SH2 or mutant GST-BPS-SH2 (E373Q) in both non-phosphorylated and phosphorylated states **(B)**, immunoblot of expressed proteins with anti-PY99 antibody and the same blot stripped and reprobed with anti-GST antibody **(C)**. The fusion proteins (12 μM) that were used in the IR kinase activity **(B)** were further examined for their effect on PTP1B activity **(D)**. Data are mean ± S.D., n = 3, **p < 0.001; *p < 0.05, P-Phosphorylated.

To determine whether Grb14 phosphorylation performs any role in IR kinase activity, we examined the effect of non-phosphorylated and phosphorylated BPS regions of Grb14 on IR kinase activity *in vitro* (Figure 1A). Non-phosphorylated and phosphorylated BPS domains of GST-Grb14 were expressed alone, or co-expressed with VSRC and purified according to the method described earlier [10]. Both inhibited the IR kinase activity equally well (Figure 1B). The crystal structure of the BPS domain revealed that a region between amino acids 373 and 381 is involved in binding to the IR, and that Glu373 is crucial for this binding [16].

The mutant GST-BPS-SH2 (E373Q) and wild-type GST-BPS-SH2 Grb14 were either expressed alone or co-expressed with VSRC [17]. The expressed proteins were purified and immunoblotted with anti-PY99 antibody. The results indicated that VSRC mediated phosphorylation of the BPS-SH2 (E373Q) and wild-type BPS-SH2 domains (Figure 1C). To ensure equal amounts of fusion proteins, we reprobed the blot with anti-GST antibody (Figure 1C).

Non-phosphorylated and phosphorylated BPS-SH2 (E373Q) domains of Grb14 were tested for their effect on IR kinase activity. This mutant BPS region failed to inhibit the IR kinase activity, regardless of phosphorylation status (Figure 1B). However, the same mutant was able to inhibit PTP1B activity, similar to the wild-type BPS region of Grb14, and the inhibition caused by the phosphorylated E373Q-BPS region was significantly greater than that of the non-phosphorylated E373Q-BPS-SH2 domain (Figure 1D). These results show that phosphorylated and non-phosphorylated BPS domains equally inhibit IR kinase activity, whereas the phosphorylated BPS domain significantly inhibits PTP1B activity to a greater extent than its non-phosphorylated counterpart. These data suggest that IR and PTP1B inhibitory activities are influenced by the BPS region of Grb14.

### Interaction of phosphorylated Grb14 with vSrc-SH2 domain

**Figure 2:**
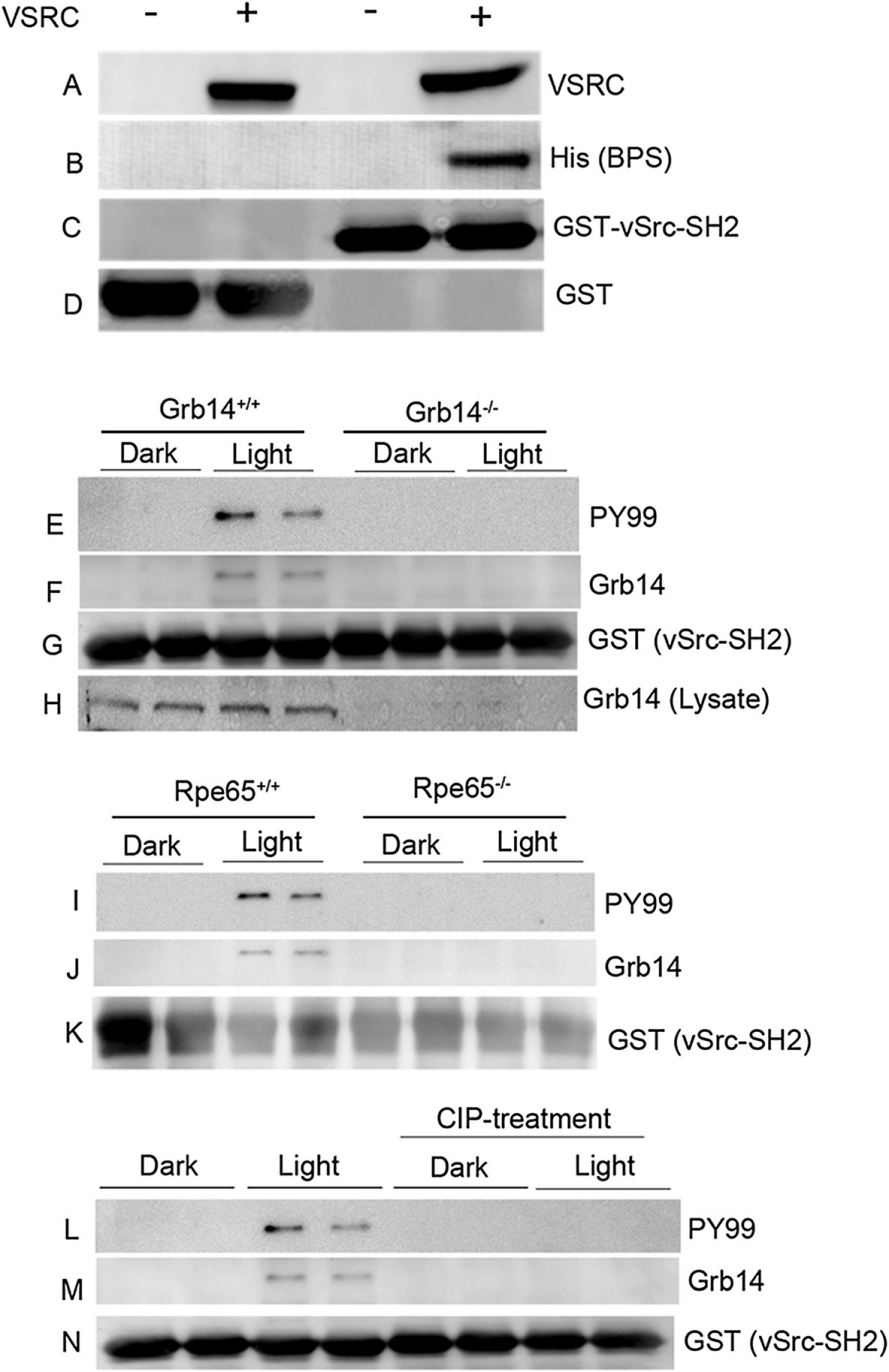
**Association of phosphorylated Grb14 with SH2 domain of vSrc *in vitro*.** The His-tagged BPS domain of Grb14 was either expressed or coexpressed with VSRC **(A)**, and the proteins were incubated with GST or GST-vSrc-SH2 fusions followed by a GST pull-down assay. The bound proteins were subjected to immunoblot analysis with the anti-His antibody **(B)** and reprobed with the anti-GST antibody **(C, D)**. The dark- and light-adapted retinal lysates from wild-type and Grb14^-/-^ mice were subjected to a GST-vSrc-SH2 pull-down assay followed by immunoblotting with anti-PY99 **(E)**, anti-Grb14 **(F)**, and anti-GST **(G)** antibodies. Wild-type and Grb14^-/-^ mouse retinal lysates were immunoblotted for Grb14 with anti-Grb14 antibody **(H)**. Retinal lysates from dark- and light-adapted wild-type and Rpe65^-/-^ mice were incubated with the GST-vSrc-SH2 fusion protein followed by a GST pull-down assay. The bound proteins were subjected to immunoblotting with anti-PY99 **(I)** and anti-Grb14 **(J)** antibodies, and reprobed with anti-GST antibody **(K)**. Retinal lysates from dark- and light-adapted wild-type mice were incubated with the GST-vSrc-SH2 fusion protein followed by GST pull-down assay. One set of the fusion was treated with calf-intestinal phosphatase for 30 min. The fusions were washed and subjected to immunoblotting with anti-PY99 **(L)** and anti-Grb14 **(M)** antibodies, and reprobed with anti-GST antibody **(N)**.

The Tyr347 residue in the BPS region of Grb14 is essential for PTP1B binding and inhibition of its activity [10]. The SH2 domain of the Src family recognizes the preferred sequence with the general motif pTyr-hydrophilic-hydrophilic-Ile/Pro [18]. The phosphorylated Grb14 BPS region has the sequence pTyr-Gln-Asn-Tyr followed by a bulky side chain of Met, which is similar to the preferred interacting sequences of the SH2 domain of Src. To investigate whether the SH2 domain of vSrc binds to the phosphorylated BPS region, the expressed His-tagged BPS region of Grb14, either alone or co-expressed with VSRC in *E. coli* (Figure 2A), was subjected to a GST pull-down assay with either GST or GST-vSrc-SH2 fusion proteins. The bound proteins were immunoblotted with anti-His antibody. The vSrc-SH2 domain associated with the BPS region only under phosphorylated conditions (Figure 2B). The blot was reprobed with anti-GST antibody to ensure equal amounts of fusion proteins in each pull-down (Figure 2C-D).

We previously reported a light-dependent phosphorylation of Grb14 *in vivo*[10]. In this study, we examined whether the vSrc-SH2 domain is able to bring down phosphorylated Grb14. We subjected the dark- and light-adapted mouse retina lysates [19] of wild-type and Grb14^-/-^ mice (Figure 2H) to a GST-vSrc-SH2 pull-down assay. The bound proteins underwent immunoblotting with anti-PY99 and anti-Grb14 antibodies (Figure 2E-F). To ensure equal amounts of fusion proteins, we reprobed the blot with anti-GST antibody (Figure 2G). The results revealed the presence of phosphorylation on Grb14 and its association with the vSrc-SH2 domain, specifically in light-adapted wild-type retinas, but not in dark-adapted wild-type retinas or in dark- and light-adapted Grb14^-/-^ mice (Figure 2E-F). These data suggest that Grb14 undergoes light-dependent phosphorylation in the retina, and that the vSrc-SH2 domain is able to associate with phosphorylated Grb14 *in vitro*. This also indicates the specificity of the interaction between Grb14 and the vSrc-SH2 domain *in vitro*.

We previously reported that Grb14 phosphorylation was absent in the retinal pigment epithelium 65 knockout (Rpe65^-/-^) mice [10]. Rpe65^-/-^ mice have opsin in their photoreceptor outer segments, but do not form photobleachable rhodopsin (the activated form), due to the absence of regeneration of the chromophore 11-*cis*-retinal [20]. To further confirm the specificity of the vSrc-SH2 domain with phosphorylated Grb14, we subjected the retinal lysates of the wild-type and Rpe65^-/-^ mice to GST-vSrc-SH2 pull-down and immunoblotting with anti-PY99 (Figure 2I) and anti-Grb14 (Figure 2J) antibodies. To ensure equal amounts of fusion proteins in each pull-down, the blot was reprobed with anti-GST antibody (Figure 2K). Loss of light-dependent phosphorylation of Grb14 occurred in the Rpe65^-/-^ mice, but Grb14 phosphorylation occurred in the wild-type mice under light-adapted conditions (Figure 2I). These results suggest that rhodopsin mediates the activation of Grb14 phosphorylation *in vivo*.

As shown in Figure 2 (panels L-N), we treated GST-vSrc pull-downs of dark- and light-adapted rat retinal lysates with calf-intestinal phosphatase (CIP) for 30 min and then washed the bound fusion proteins twice in wash buffer [21] prior to immunoblot analysis. The results indicate a loss of PY99 immunoreactivity and a loss of Grb14 association with vSrc-SH2 domain in CIP-treated retinas compared to light-adapted CIP-untreated retinas (Figure 2L-N). This experiment further confirms that Grb14 undergoes tyrosine phosphorylation *in vivo* and that phosphorylation is necessary for its association with the vSrc-SH2 domain *in vitro*. The vSrc-SH2 domain could be used as a tool to study the phosphorylation status of Grb14 in various tissues and cell types. Currently, there are no species-specific Grb14 antibodies with which to perform immunoprecipitation. The described vSrc-SH2 pull-down assay would be an excellent choice to study Grb14 phosphorylation under physiological and pathological conditions. The second advantage of this approach is that mouse retinas provide a small amount of tissue material: the pull-down assay would be of great advantage in studying Grb14 phosphorylation. However, we failed to observe an endogenous interaction between phosphorylated Grb14 and Src in mammalian cells (data not shown).

### Association of Src and Grb14 to light-adapted outer segment membranes

**Figure 3:**
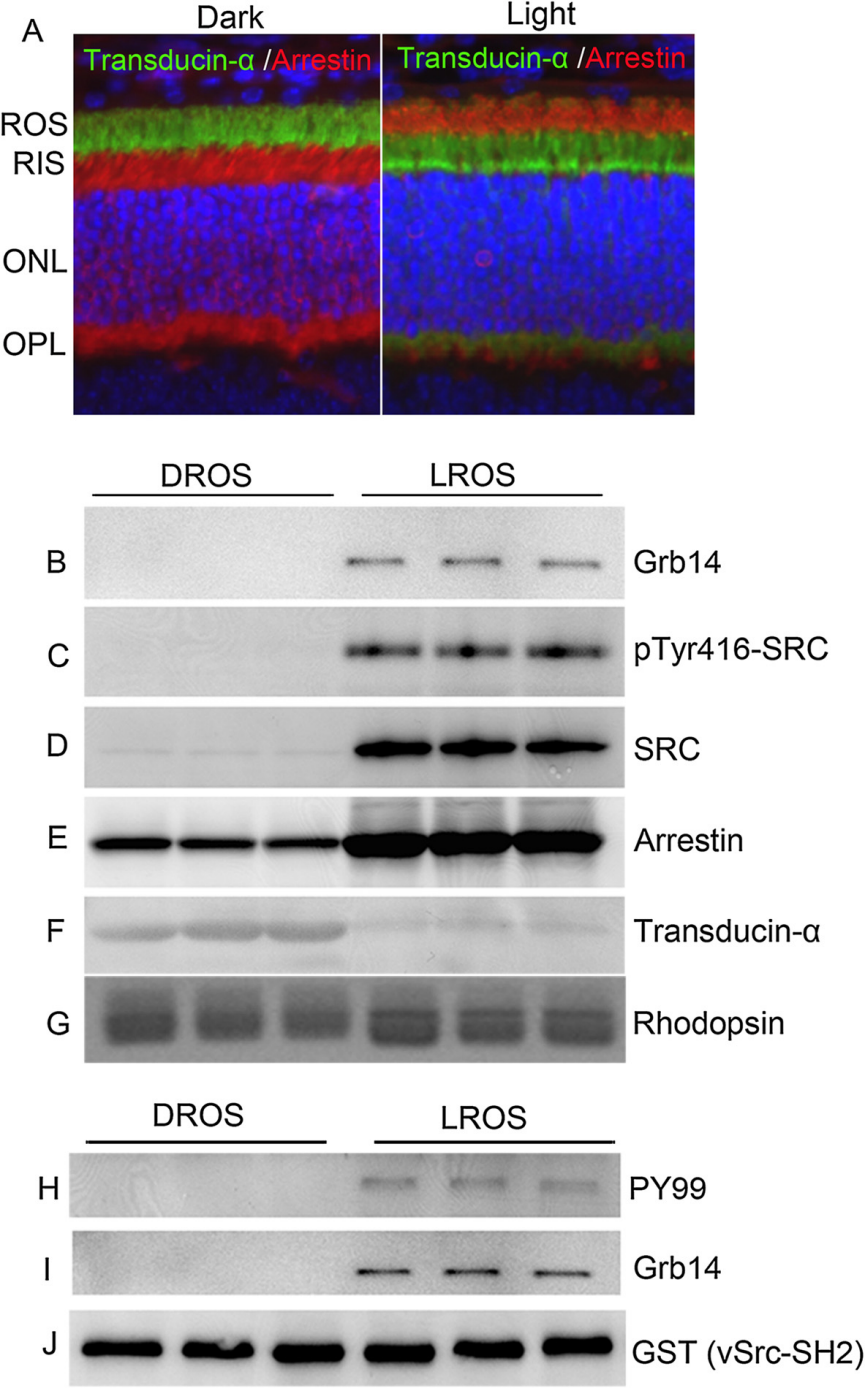
**Association of Grb14 and Src to light-adapted rod outer segment membranes.** Immunofluorescence analysis with anti-transducin (Tα) and anti-arrestin antibody was performed with dark- and light-adapted (300 lux for 30 min) rat retinal sections **(A)**. The images are of the same section viewed with a filter to detect transducin α (green), arrestin (red), and DAPI stained nuclei (blue). ROS, rod outer segment; RIS, rod inner segment; ONL, outer nuclear layer; OPL, outer plexiform layer. ROS membranes were prepared from dark- and light-adapted rats on a discontinuous sucrose density gradient centrifugation. DROS and LROS proteins were subjected to immunoblot analysis with anti-Grb14 **(B)**, anti-pTyr416-Src **(C)**, anti-Src **(D)**, anti-arrestin **(E)**, anti-transducin-α **(F)**, and anti-rhodopsin **(G)** antibodies. Solubilized DROS and LROS proteins were incubated with the GST-vSrc-SH2 fusion protein for 2 h followed by GST pull-down assay. The bound proteins were subjected to immunoblot analysis with anti-PY99 **(H)**, anti-Grb14 **(I)**, and anti-GST **(J)** antibodies.

We previously reported a light-induced tyrosine phosphorylation of Grb14 by non-receptor tyrosine kinase Src [10]. Src activation has been shown to occur in photoreceptor outer segments [22]. In our previous study, we carried out the published experiments with total retinal lysates. In this experiment, we examined the state of tyrosine phosphorylation on Grb14 and Src in rod outer segments (ROS) prepared from dark- and light-adapted rats. ROS membranes were prepared from dark- (DROS) and light-adapted (LROS) rats on a discontinuous sucrose density gradient centrifugation. The adaptability of animals to dark and light conditions was examined with rod arrestin and rod transducin alpha (Tα) immunolocalization. In dark-adapted retinas, arrestin is localized to the rod inner segments and the outer plexiform layer (Figure 3A), while Tα is localized to rod outer segments (Figure 3A). Upon light illumination, arrestin is translocated to photoreceptor outer segments, while Tα moves to rod inner segments (Figure 3A).

Three independent preparations of DROS and LROS proteins were subjected to immunoblot analysis with Grb14, pTyr416-Src, Src, arrestin, Tα, and rhodopsin. The results indicate that photoreceptor marker arrestin is enriched in LROS (Figure 3E), while Tα is enriched in DROS (Figure 3F), further confirming the strict adaptability of rats to dark- and light-adaptation. The integral membrane protein opsin expression remains the same between DROS and LROS (Figure 3G). We found the association of both Grb14 and Src with LROS, but not with DROS (Figure 3B and D). The pTyr416-Src blot shows that LROS-associated Src is tyrosine phosphorylated (Figure 3C).

To determine whether phosphorylation of Grb14 occurs in LROS, we subjected the solubilized DROS and LROS to a GST-vSrc-SH2 pull-down assay. The bound proteins were immunoblotted with anti-PY99 and anti-Grb14 antibodies (Figure 3H and 3I). To ensure equal amounts of fusion proteins, we reprobed the blot with anti-GST antibody (Figure 3J). The results revealed the presence of phosphorylation on Grb14 and its association with the vSrc-SH2 domain, specifically in LROS (Figure 3H). These data suggest that Grb14 undergoes light-dependent phosphorylation in the retina, and that the vSrc-SH2 domain is able to associate with phosphorylated Grb14 *in vitro*. These experiments suggest that tyrosine phosphorylation of Src and Grb14 occurs in outer segments upon light-adaptation.

### Spatial and temporal regulation of IR activation and interplay of PTP1B and Grb14

**Figure 4:**
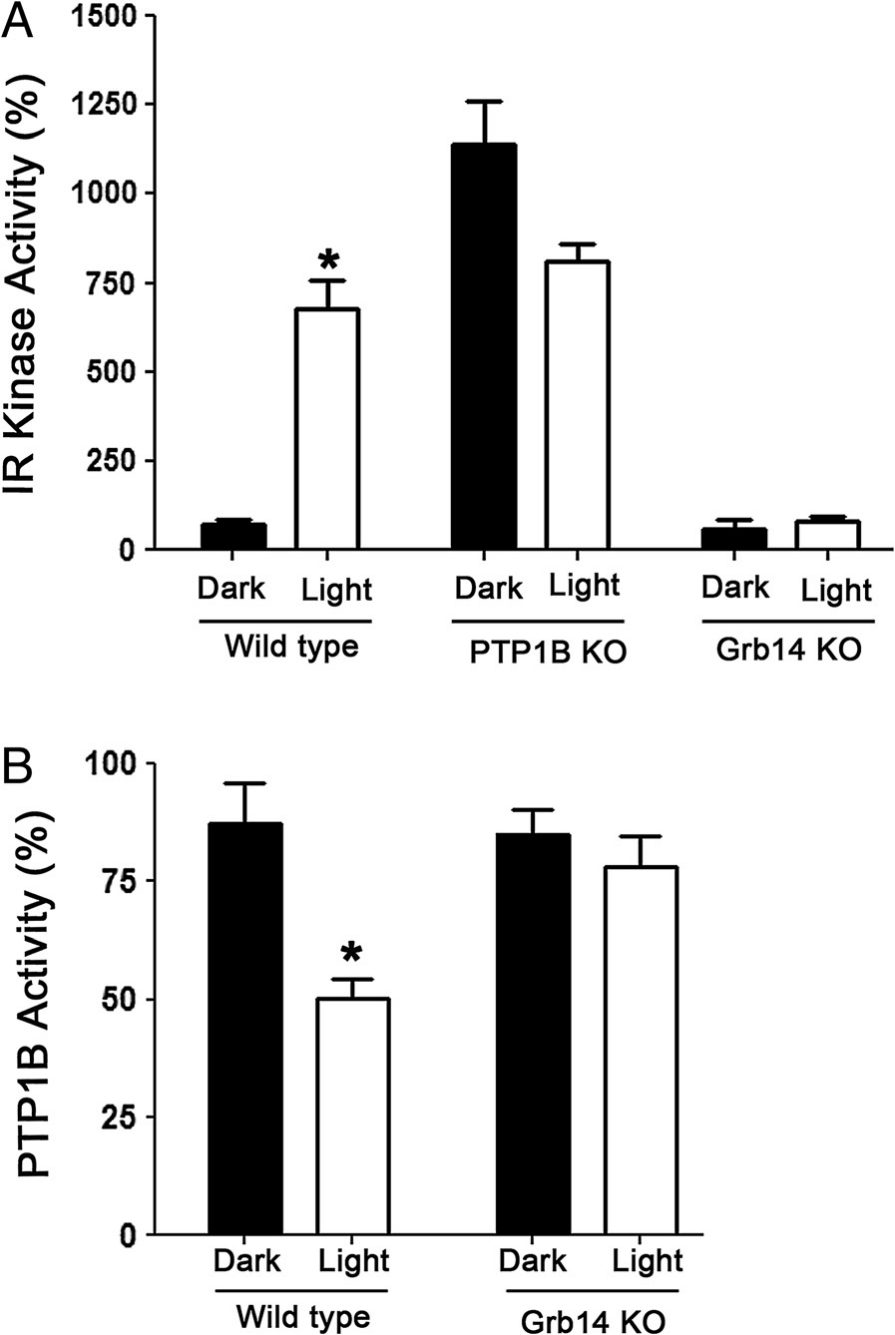
**Regulation of IR activation through PTP1B and Grb14.** In support of our current findings, Figure 4 presents aggregate data based on the results from our previous studies of the IR kinase and PTP1B activities in wild-type, PTP1B KO, and Grb14 KO mice [8,10,19]. The IR immunoprecipitates from dark- and light-adapted wild-type, PTP1B^-/-^, and Grb14^-/-^ mice were subjected to IR kinase activity employing poly Glu:Tyr peptide as a substrate **(A)**. PTP1B activity was measured in retinas harvested from the dark- and light-adapted wild-type and Grb14^-/-^ mice **(B)**. Data are mean ± SD, n = 5, *p < 0.05. The IR kinase activity and PTP1B activity in dark-adapted retinas was set at 100%.

In support of our current findings, Figure 4 presents aggregate data based on the results from our previous studies of the IR kinase and PTP1B activities in wild-type, PTP1B KO, and Grb14 KO mice [8,10,19]. Using this data, we summarized the spatial and temporal regulation of IR activation and interplay of PTP1B and Grb14 (Figure 4). We detected a light-dependent IR kinase activity in wild-type mice (Figure 4A). In the absence of PTP1B, IR kinase activity in dark-adapted retinas was similar to the IR kinase activity found in light-adapted wild-type mouse retinas (Figure 4A). In Grb14^-/-^ mice, the light-dependent activation of IR kinase activity was lost and was comparable to the dark-adapted retinal controls (Figure 4A). Consistent with these observations, PTP1B activity was significantly lower in the light-adapted retinas than in the dark-adapted wild-type controls (Figure 4B). The loss of light-dependent inhibition of PTP1B activity in Grb14^-/-^ mice demonstrates that Grb14 regulates light-dependent PTP1B activity (Figure 4B). These results suggest that IR activation is mediated through PTP1B inhibition via Grb14.

### Grb14 phosphorylation is specific to retina

**Figure 5:**
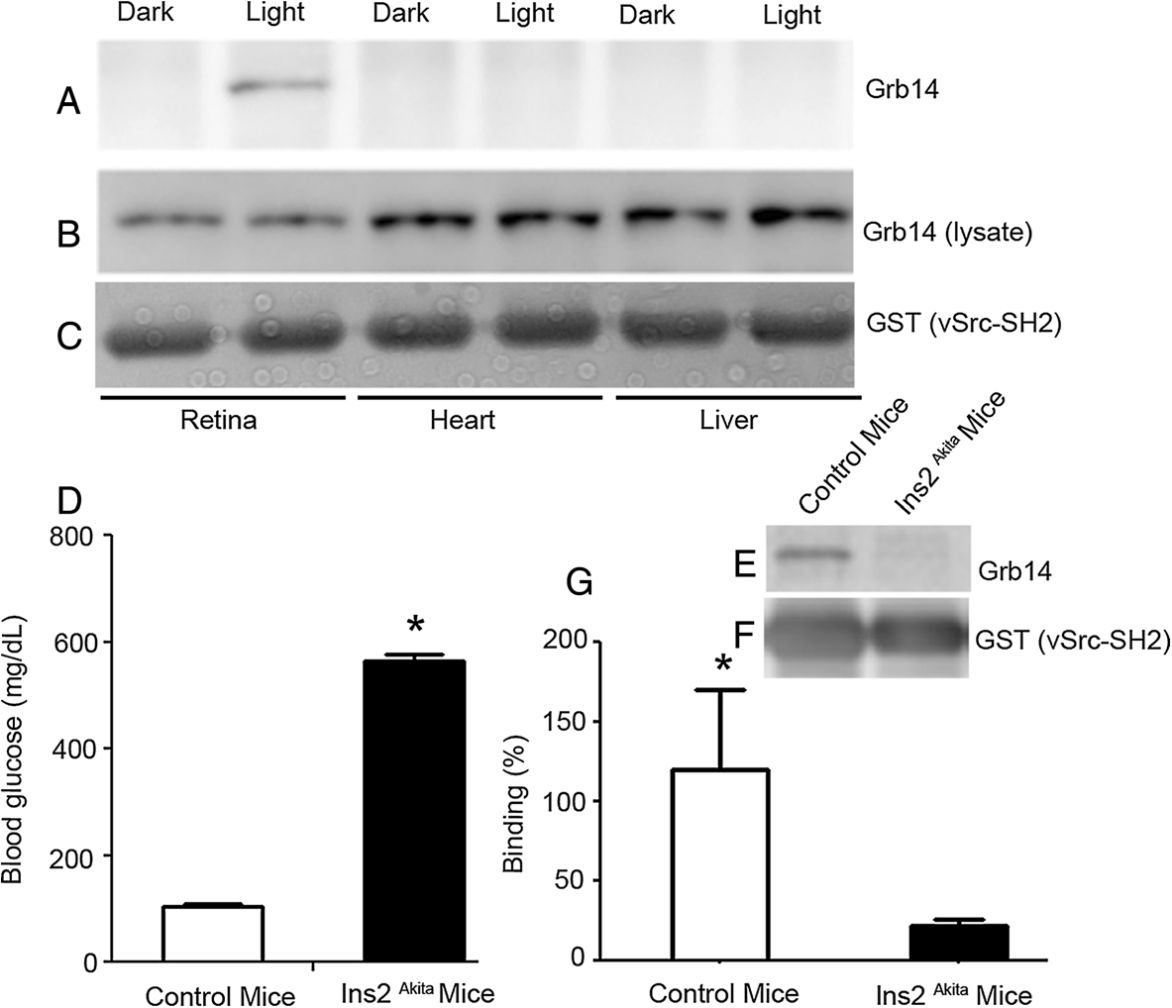
**Tissue specific phosphorylation of Grb14.** Retinal, heart, and liver protein lysates from dark- and light-adapted rats were incubated with the GST-vSrc-SH2 fusion protein, followed by GST pull-down assay. The bound proteins were subjected to immunoblotting with anti-Grb14 **(A)** and anti-GST **(C)** antibodies. Tissue lysates underwent immunoblot analysis with anti-Grb14 antibody **(B)**. Blood was drawn from control and Ins2^Akita^ mouse tail veins, and the blood glucose levels were monitored with a glucometer **(D)**. Data are mean ± SE, n = 8, *p < 0.001. Control and Ins2^Akita^ mouse retinal lysates were subjected to GST-vSrc-SH2 fusion protein, followed by GST pull-down assay. The bound proteins were subjected to immunoblot analysis with anti-Grb14 **(E)** and anti-GST **(F)** antibodies. Densitometric analysis of 4 independent immunoblots of Grb14 was performed in the linear range of detection and absolute values were then normalized to v-Src-SH2 **(G)**. Values are mean ± SEM, (n = 4), *p < 0.05. The binding of Grb14 to the v-Src-SH2 domain in control mice was set at 100 percent.

We isolated retinal, heart, and liver tissues from dark- and light-adapted rats, and the respective lysates were incubated with vSrc-SH2 fusion followed by a GST pull-down assay. The bound proteins were subjected to immunoblotting with anti-Grb14 antibody (Figure 5A) and the blot was reprobed with anti-GST antibody (Figure 5C). Tissue lysates also underwent immunoblot analysis with anti-Grb14 antibody, to examine the expression of Grb14 in all of the tissues. Even though Grb14 was expressed in all tissues (Figure 5B), the phosphorylation of Grb14 was exclusively observed in retinal tissue under light-adapted conditions (Figure 5A).

### Decreased Grb14 phosphorylation in type 1 diabetic mouse retina

In this study, we examined the phosphorylation status of Grb14 in type 1 diabetic Ins2^Akita^ mice. The Ins2^Akita^ mutation results in a single amino acid substitution in the insulin 2 gene that causes misfolding of the insulin protein [23]. Male mice heterozygous for this mutation have progressive loss of beta-cell function, decreased pancreatic beta-cell density, and significant hyperglycemia as early as 4 weeks of age [23]. The mean blood glucose levels of control and Ins2^Akita^ mice are shown in Figure 5D. We considered mice with blood glucose levels higher than 250 mg/dL to be hyperglycemic. Control and Ins2^Akita^ mouse retinal lysates underwent GST pull-down assays with the vSrc-SH2 domain, followed by immunoblot analysis with anti-Grb14 antibody (Figure 5E). The blot was reprobed with anti-GST antibody (Figure 5F). The results indicate a reduced binding of Grb14 with the vSrc-SH2 domain in Ins2^Akita^ mouse retinas compared to controls (Figure 5G), suggesting that Grb14 phosphorylation is reduced in type 1 diabetic mouse retinas.

## Discussion

Our experiments show that the phosphorylation status of Grb14 is the key element in determining whether it will execute a negative or positive role in IR signaling. We found no significant difference in the inhibition of IR kinase activity when we compared phosphorylated and non-phosphorylated BPS regions of Grb14 *in vitro* (Figure 1B). However, phosphorylated Grb14 produced greater inhibition of PTP1B activity than its non-phosphorylated form (Figure 1D). We also established that Grb14 undergoes a light-dependent tyrosine phosphorylation *in vivo*, through light-dependent activation of a non-receptor tyrosine kinase Src. The mechanism of light-dependent activation of Src is not known. However, it has previously been shown that Src associates with bleached opsin [22]. Src-homology phosphotyrosyl phosphatase 2 (Shp2) is known to regulate the Src family kinases by dephosphorylating Tyr-527 on the C-terminus of Src, thereby activating it [24]. Shp2 is expressed in photoreceptors [25]. Additionally, we found a light-dependent activation of Shp2 in the retina (data not shown). We are currently conducting studies to examine this intriguing phenomenon.

Our previous studies suggest that PTP1B resides in rod outer segment membranes, and dephosphorylates the IR in dark-adapted retinas, thus inactivating the IR [19]. Grb14 is localized to rod inner segments in dark-adapted retinas [8]. Following photon capture by rhodopsin, Grb14 moves to rod outer segments, where it is tyrosine phosphorylated by light-activated Src and binds to PTP1B: binding inhibits PTP1B phosphatase activity and thus activates the IR [10].

**Figure 6:**
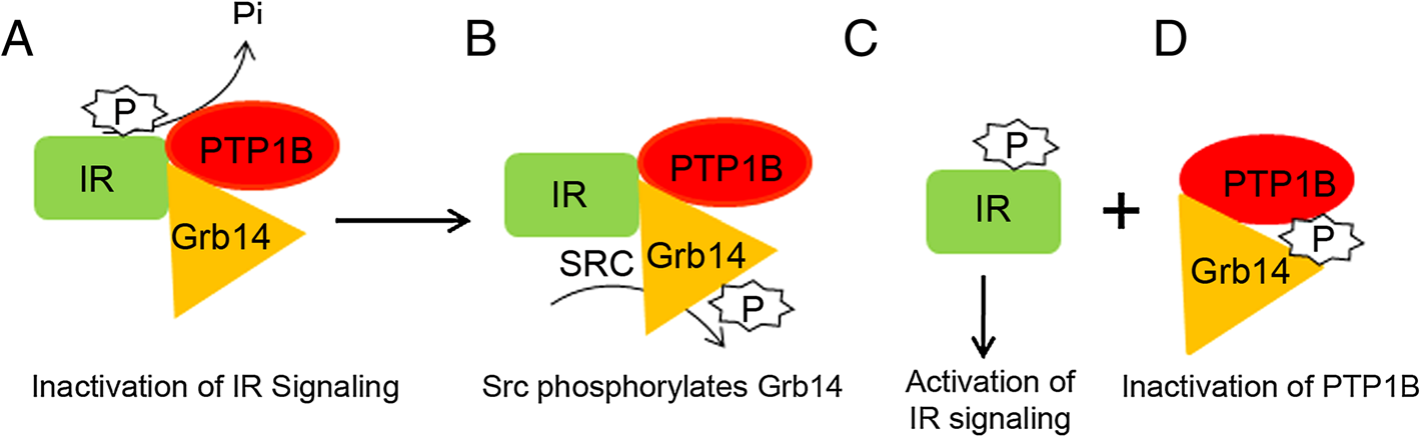
**Activation of IR signaling in the retinal neurons.** IR is inactivated through dephosphorylation by PTP1B and a direct inhibition of IR kinase activity by Grb14 **(A)**. Src kinase phosphorylates Grb14 **(B)**, which triggers the affinity switch from IR towards PTP1B, where it binds and directly inhibits the phosphatase activity **(D)**. The IR, free from Grb14 and PTP1B, thus regulates the downstream neuron survival pathway **(C)**. IR, insulin receptor; PTP1B, protein tyrosine phosphatase 1B; Grb14, growth factor receptor-bound protein 14; P, phosphorylation; Pi, inorganic phosphate.

Results from our earlier research also showed that ablation of Grb14 increases PTP1B activity, which results in decreased IR activation [8,10]. Based on our previous data and the results of the current study, we propose a model explaining how Grb14 performs a negative or positive role in IR signaling (Figure 6). In tissues in which Grb14 does not undergo phosphorylation, Grb14 can directly interact with the IR and acts as a negative regulator. In tissues in which Grb14 undergoes tyrosine phosphorylation, Grb14 binds to PTP1B and inhibits its activity, thereby promoting IR signaling. In the event of the loss of phosphorylation, the non-phosphorylated form of Grb14 can act as a negative regulator and downregulate IR signaling. Consistent with this hypothesis, we found decreased phosphorylation of Grb14 in diabetic type 1 Ins2^Akita^ mouse retinas (Figure 5E-G). Decreased retinal IR activation has previously been reported in this mouse line [26]. Also, decreased IR kinase activity in retinal cells has been reported in experimental and genetic models of type 1 diabetes [26,27]. The decreased IR activation is due to increased retinal PTP1B activity [27]. In Rpe65^-/-^ mice, a mouse model for defective rhodopsin activation [20], the lack of phosphorylation of Grb14 also enhanced PTP1B activity [10,19]. These studies suggest that decreased IR kinase activity [26] could be due to a defect in the phosphorylation of Grb14. In summary, our results suggest that the phosphorylation status of the BPS region of Grb14 determines whether Grb14 plays a positive or negative role in IR signaling. Further, activators of Grb14 phosphorylation may have therapeutic potential in protecting the dying retinal neurons in retinal degenerative diseases.

## Materials and methods

PTP1B substrate RRLIEDAE_P_YAARG and the phosphatase assay reagents were obtained from Upstate Biotechnology (Lake Placid, NY). The monoclonal PY99 antibody was procured from Santa Cruz Biotechnology (Santa Cruz, CA). A quick-change, site-directed mutagenesis kit was purchased from Stratagene (La Jolla, CA). Anti-His, anti-Src, and anti-Phospho Src (pTyr 416) antibodies were acquired from Cell Signaling Technology, Inc. (Beverly, MA). Anti-glutathione S-transferase (GST) antibody and glutathione-Sepharose 4B matrix were obtained from Amersham Biosciences Corp. (Piscataway, NJ). Human recombinant-insulin receptor kinase-GST fusion was acquired from Calbiochem (San Diego, CA). [γ^32^ P] ATP was purchased from New England Nuclear (Boston, MA). Generation of a polyclonal Grb14 antibody was described earlier [8]. All other reagents were of analytical grade from Sigma (St. Louis, MO).

### Animals

All animal work was performed in strict accordance with the Association for Research in Vision and Ophthalmology’s statement on the “Use of Animals in Ophthalmic and Vision Research”. All protocols were approved by the Institutional Animal Care and Use Committee of the University of Oklahoma Health Sciences Center and the Dean A. McGee Eye Institute. A breeding colony of albino Sprague-Dawley (SD) rats is maintained in our vivarium in cyclic light (5 lux; 12 h on/12 h off). Experiments were carried out on both male and female rats (150–200 g). Grb14^-/-^ mice were kindly provided by Dr. Roger J Daly (Garvan Medical Institute, Sydney, Australia) and Rpe65^-/-^ mice were provided by Dr. Jian-Xing Ma (University of Oklahoma, USA). The generation of rod specific PTP1B knockout animals has been reported previously [19]. C57BL/6J Ins2^Akita^ heterozygote mice (Jackson Laboratory, Bar Harbor, ME) were bred in the Dean A. McGee Eye Institute animal vivarium. Diabetic phenotype and genotype were confirmed 4.5 weeks after birth by blood glucose >250 mg/dL (TrueTrack Smart System; AR-MED Ltd, Egham, UK) in a drop of blood from a tail puncture. The disease is 100% penetrant in mice with the Ins2 mutation [28].

### Insulin receptor kinase assay

The phosphorylation reaction was performed essentially as described [8] in a total volume of 25 μL of 50 mM Tris-HCl buffer (pH 7.0), 50 mM MgCl_2_, 5 mM MnCl_2_, 50 mM Na_3_VO_4_, 7 μg/mL *p-*nitrophenyl phosphate, 3 mg/mL poly Glu:Tyr peptide, IR cytoplasmic domain (1 μg), and Grb14 BPS fusion protein (either phosphorylated or non-phosphorylated). The reaction was initiated by adding 2.5 μL [γ^32^P] ATP to reach a final concentration of 200 μM, and the reaction was terminated by adding 10 μL of 50% (v/v) acetic acid. Twenty-five microliters of assay mixture was spotted onto P81 phosphocellulose filter paper disks (1.5 cm × 1.5 cm), which were immersed in a solution containing 0.75% phosphoric acid (v/v). The filter paper disks containing the bound phosphorylated peptide were washed three times with phosphoric acid and rinsed in acetone. Radioactivity was quantified in 5 mL of liquid scintillation cocktail and counted (Ready Safe Liquid Scintillation Cocktail and Liquid Scintillation Counter, Beckman, Fullerton, CA).

### PTP1B activity assay

The *in vitro* PTP1B activity assay was conducted based on a previously published protocol using the peptide RRLIEDAE_P_YAARG (Upstate Biotechnology) [10]. The reaction was carried out in 60 μL volume in PTP assay buffer [100 mm HEPES (pH 7.6), 2 mM EDTA, 1 mm dithiothreitol, 150 mm NaCl, and 0.5 mg/ml bovine serum albumin] at 30°C. At the end of the reaction, 40 μL aliquots were placed into a 96-well plate, 100 μL of Malachite Green Phosphatase reagent (Upstate Biotechnology) were added, and absorbance was measured at 630 nm.

### Plasmid constructs

The amino acid sequences corresponding to the BPS region of Grb14 (amino acids 342 - 438) and the BPS-SH2 (amino acids 342 - 540) region were amplified by polymerase chain reaction (PCR) and cloned into pGEX-4T-1 GST fusion vector. The BPS region of Grb14 was also cloned into pTrcHisA vector. Cloning and expression of retinal PTP1B was described earlier [27]. The SH2 region (amino acids 151-250) of VSRC was amplified with sense, GGA TCC GGG AAG ATC ACT CGT CGG GAG TCC, antisense, GAA TTC CTA GGG CTT GGA CGT GGG GCA GAC, primers. After sequencing, the fragment was excised as *BamHI/EcoRI* fragment and ligated into pGEX-2TK vector. The sequence of each clone was verified by DNA sequencing. All inductions yielded proteins of the expected size as determined by Coomassie staining. The phosphorylation of each fusion protein in *E. coli* (BL21) was achieved by coexpressing a tyrosine kinase VSRC under the control of different replicons [17]. The phosphorylated and non-phosphorylated GST fusion proteins were purified using glutathione-Sepharose beads.

### Site-directed mutagenesis

Site-directed mutagenesis (SDM) was carried out according to the method described earlier [29]. The reaction mixture contained SDM buffer [200 mM Tris-HCl (pH 8.8), 100 mM KCl, 100 mM (NH_4_)_2_SO_4_, 20 mM MgSO_4_, 1% Triton X-100, 1 mg/ml nuclease-free bovine serum albumin, 1 mM deoxynucleotide mix (dATP, dCTP, dTTP, and dGTP)], 50 ng of vector, BPS-SH2 (R466A), and 125 ng of sense and antisense primers with mutations in a total volume of 50 μl, followed by the addition of 2.5 units of pfu DNA polymerase. The primers used in the site-directed mutagenesis are as follows: E373Q (sense, AGA AGT ATA TCA CAG AAT TCC CTG GTA GCA; antisense, TGC TAC CAG GGA ATT CTG TGA TAT ACT TCT). The sequence of each mutation was verified by DNA sequencing. The phosphorylation of each fusion protein in *E. coli* (BL21) was achieved by coexpressing a tyrosine kinase VSRC under the control of different replicons [17]. The phosphorylated and non-phosphorylated GST fusion proteins were purified using glutathione-Sepharose beads.

### GST pull-down assays

Pull-down experiments were carried out as described using GST fusion proteins that had been adsorbed onto a glutathione - Sepharose 4B matrix. Retinal lysates were incubated overnight with either GST or GST fusion proteins at 4°C with continuous stirring. The Sepharose beads were washed three times in 500 μL of PBS and centrifuged at 5000 rpm for 30 - 60 s at 4°C. Bound proteins were eluted by boiling in 2X SDS sample buffer for 5 min before separating using 10% SDS - PAGE. The gels were then subjected to immunoblot analysis with appropriate antibodies.

### Preparation of rat rod outer segments

Rod outer segments (ROS) were prepared from rat retinas using a discontinuous sucrose gradient centrifugation as previously described [30]. Retinas were homogenized in 4.0 ml of ice-cold 47% sucrose solution containing 100 mM NaCl, 1 mM EDTA, 1mM NaVO_3_, 1 mM PMSF, and 10 mM Tris-HCl (pH 7.4) (buffer A). Retinal homogenates were transferred to 15-ml centrifuge tubes and sequentially overlaid with 3.0 ml of 42%, 3.0 ml of 37%, and 4.0 ml of 32% sucrose dissolved in buffer A. The gradients were spun at 82,000 × g for 1 h at 4°C. The 32/37% interfacial sucrose band containing ROS membranes was harvested and diluted with 10 mM Tris-HCl (pH 7.4) containing 100 mM NaCl and 1 mM EDTA, and centrifuged at 27,000 × g for 30 min. The ROS pellets were resuspended in 10 mM Tris-HCl (pH 7.4) containing 100 mM NaCl and 1 mM EDTA, and stored at –20°C. All protein concentrations were determined by the BCA reagent following the manufacturer’s instructions.

### SDS-page and immunoblotting

Proteins were resolved by 10% SDS-PAGE and transferred to nitrocellulose membranes. The blots were washed twice for 10 min with TTBS [20 mM Tris-HCl (pH 7.4), 100 mM NaCl, and 0.1% Tween-20] and blocked with either 5% bovine serum albumin or non-fat dry milk powder (Bio-Rad) in TTBS for 1 h at room temperature. Blots were then incubated with anti-Grb14 (1:1000), anti-pTyr-416-Src (1:1000), anti-Src (1:1000), anti-His (1:1000), anti-PY99 (1:1000), anti-arrestin (1:1000), anti-Tα (1:1000), at 4°C overnight or anti-GST (1:5000), anti-opsin (1:10,000) for 1 h at room temperature. Following primary antibody incubations, immunoblots were incubated with HRP-linked secondary antibodies (mouse or rabbit) and developed by enhanced chemiluminescence according to the manufacturer’s instructions.

### Statistical methods

Data were analyzed and graphed using GraphPad Prism software (GraphPad Software, San Diego, CA). The data were expressed as the mean ± S.E. and compared by Student's *t* test for unpaired data. The significance level was set at *p* < 0.05 or *p* < 0.001.

## Abbreviations

Grb14: Growth factor receptor-bound protein 14; PTP1B: Protein tyrosine phosphatase 1B; Shp2: Src-homology phosphotyrosyl phosphatase 2; IR: Insulin receptor.

## Competing interests

The authors declare that they have no competing interests.

## Authors’ contributions

Project planning, data analysis, and manuscript composition were performed by RR. Experimental work was performed by RR, DKB and AR. All authors read and approved the final manuscript.
